# Appel-reagent-mediated transformation of glycosyl hemiacetal derivatives into thioglycosides and glycosyl thiols

**DOI:** 10.3762/bjoc.9.112

**Published:** 2013-05-22

**Authors:** Tamashree Ghosh, Abhishek Santra, Anup Kumar Misra

**Affiliations:** 1 Bose Institute, Division of Molecular Medicine, P-1/12, C.I.T. Scheme VII-M, Kolkata-700054, India, Fax: 91-33-2355 3886

**Keywords:** Appel reagent, carbon tetrabromide, glycosyl hemiacetal, glycosyl thiol, thioglycoside, triphenylphosphine

## Abstract

A series of glycosyl hemiacetal derivatives have been transformed into thioglycosides and glycosyl thiols in a one-pot two-step reaction sequence mediated by Appel reagent (carbon tetrabromide and triphenylphosphine). 1,2-*trans*-Thioglycosides and β-glycosyl thiol derivatives were stereoselectively formed by the reaction of the in situ generated glycosyl bromides with thiols and sodium carbonotrithioate. The reaction conditions are reasonably simple and yields were very good.

## Introduction

**Scheme 1:**
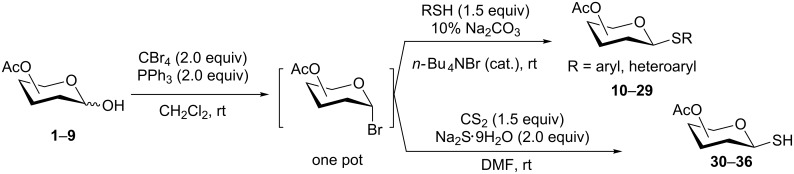
Appel-reagent-mediated transformation of glycosyl hemiacetal derivatives into thioglycosides and β-glycosyl thiol derivatives in one pot.

Thioglycosides (1-thiosugar) are widely used glycosyl donors in glycosylation reactions [1–5]. Due to their thermal and chemical stability, they have been used as stable intermediates for functional-group transformations as well as stereoselective glycosylations. Thioglycosides can be transformed into various other glycosyl donors [6–10] (e.g., sulfoxide, sulfone, fluoride, bromide, hemiacetal, etc.) and hence the thio functionality is often used as a temporary anomeric protecting group. Thioglycosides can act as a glycosyl donor as well as glycosyl acceptor depending on the reaction conditions (orthogonal glycosylations) [11–12]. Due to their stability towards enzymatic hydrolysis, several thioglycosides have been evaluated as enzyme inhibitors [13–14]. As a consequence a large number of reports have appeared in the past for the preparation of thioglycosides. Conventionally, thioglycosides are prepared by the reaction of glycosyl acetates with thiols or trimethylsilylthiols in the presence of a Lewis acid (borontrifluoride diethyletherate, stannic chloride, trimethylsilyl trifluoromethanesulfonate, etc.) [15–20]. Other methods for the synthesis of thioglycosides include (a) reduction of disulfides using metallic salts [21–22] or nonmetallic reducing agents (triphenylphosphine or combination of triethylsilane and BF_3_·OEt_2_) followed by the reaction of the in situ generated thiolate ions with glycosyl bromides under phase-transfer conditions [23] or in ionic liquids [24]; (b) reaction of glycosyl bromides with thiols under phase-transfer conditions [25]; and (c) conversion of glycosyl acetates and bromides to isothiouronium salts followed by hydrolytic alkylation of isothiouronium salts with alkyl halide in the presence of a base [26–27]. Most of the reactions have several shortcomings, which include formation of an anomeric mixture of the products, instability of the starting materials (glycosyl bromides, etc.), multiple steps, unsatisfactory yields, use of metallic salts, use of expensive reagents, pregeneration of glycosyl isothiouronium salts, etc. Similar to thioglycosides, glycosyl thiol derivatives are useful intermediates for the synthesis of various thiooligosaccharides, glycoproteins and glycolipids [28–32]. The anomeric configurations of glycosyl thiols mostly remain unaffected in comparison to the glycosyl hemiacetal derivatives during their synthetic transformations [4]. Glycosyl thiol derivatives act as precursors for the preparation of several glycosyl donors such as thioglycosides [33–34], glycosyl sulfenamides [35] and sulfonamides [36], glycosyl disulfides [37], glycosyl thionolactones [38], etc. A number of reports are available for the preparation of glycosyl thiols, which include (a) a two-step reaction of glycosyl halide or acetate with thiourea or thioacetate and hydrolysis of the resulting intermediates [26–27]; (b) reaction of hydrogen sulfide gas with glycosyl halides in hydrogen fluoride [39]; (c) treatment of the glycosyl hemiacetal derivatives with Lawesson’s reagent [40] and (d) treatment of 1,6-anhydro sugar derivative [41] and glycosyl trichloroacetimidate derivatives [42] with bis(trimethylsilyl) sulfide. However, most of the reactions have several inherent shortcomings, such as the use of reactive starting materials, longer reaction time, multiple steps, unsatisfactory yield, formation an isomeric mixture, use of expensive reagents, use of hazardous gases, etc. Therefore, the development of convenient reaction conditions for the stereoselective preparation of thioglycosides and glycosyl thiols that are nonhazardous is pertinent. Recently, we reported the stereoselective preparation of β-glycosyl thiol derivatives by the treatment of glycosyl bromide derivatives with the in situ generated sodium carbonotrithioate (a combination of CS_2_ and Na_2_S·9H_2_O) [43]. Although, the reaction is highly stereoselective and high yielding it involves the handling of unstable glycosyl bromide. Therefore, as an extension of the earlier report [43], it was envisioned that the treatment of a stable glycosyl hemiacetal derivative with Appel reagent (carbon tetrabromide (CBr_4_) and triphenylphosphine (PPh_3_)) [44] could generate the glycosyl bromide in situ, [45–46] which, on reaction with thiol or sodium carbonotrithioate (generated in situ from CS_2_ and Na_2_S·9H_2_O) [43] in one-pot, could furnish thioglycosides and glycosyl thiol derivatives stereoselectively. We report herein, our findings on the Appel-reagent-mediated transformation of glycosyl hemiacetal derivatives to thioglycosides and glycosyl thiol derivatives (Scheme 1).

## Results and Discussion

Initially, 2,3,4,6-tetra-*O*-acetyl-α,β-D-glucopyranose (**1**; 1.0 mmol) was treated with a mixture of CBr_4_ (1.5 mmol) and PPh_3_ (1.5 mmol) in CH_2_Cl_2_ (5 mL) at room temperature. The starting material was consumed in 6 h to give a faster-moving product (acetobromo-α-D-glucose). To the reaction mixture were added thiophenol (1.0 mmol), 10% aq Na_2_CO_3_ (5 mL) and tetrabutylammonium bromide (TBAB, catalytic) and the reaction mixture was stirred at room temperature for another 4 h. Aqueous work up of the biphasic reaction mixture furnished phenyl 2,3,4,6-tetra-*O*-acetyl-1-thio-β-D-glucopyranoside (**10**) in 80% yield. After a series of experimentation it was observed that the use of 2.0 equiv each of CBr_4_ and PPh_3_ led to the full consumption of compound **1** in 4 h, and addition of 1.5 equiv thiophenol and 10% aq Na_2_CO_3_ under phase-transfer reaction conditions led to the formation of compound **10** in 88% yield in an additional 4 h (Scheme 1). The reaction was carried out in a set of commonly used organic solvents (e.g., toluene, EtOAc, CH_3_OH, CH_3_CN, THF, DMF, DMSO, etc.) and it was observed that the reaction proceeds smoothly in CH_2_Cl_2_ and DMF in a similar fashion to the first step. Since CH_2_Cl_2_ is a low-boiling-point solvent and can be used directly in the next step, this solvent was chosen in the first step of the reaction (Table 1). Increasing the quantity of the reagents (CBr_4_ and PPh_3_) did not change the reaction time and yield significantly. However, reducing the quantity of reagents led to a low yield of conversion in a longer reaction time (Table 2). Under the optimized reaction conditions a series of 1,2-*trans*-thioglycosides were prepared from various glycosyl hemiacetals in excellent yield (Table 3). A large-scale (5 g) preparation of compound **10** was also achieved under the reaction conditions in similar yield.

**Table 1:** Screening of solvents for the in situ conversion of glycosyl hemiacetal to glycosyl bromide.

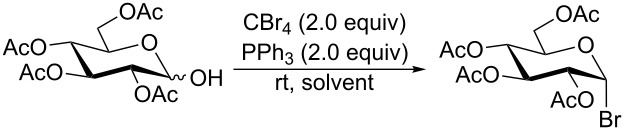			

Entry	Solvent	Time (h)	Conversion (%)

1	CH_2_Cl_2_	4	90
2	CH_3_CN	10	40^a^
3	THF	10	40^a^
4	DMF	4	85
5	EtOAc	12	20^a^
6	Toluene	12	10^a^
7	DMSO	5	80
8	CH_3_OH	4	–^b^

^a^The rest of the starting material remained unreacted; ^b^the starting material was degraded.

**Table 2:** Optimization of the in situ conversion of glycosyl hemiacetal to glycosyl bromide.

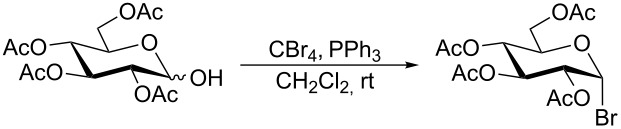				

Entry	CBr_4_ (equiv)	PPh_3_ (equiv)	Time (h)	Conversion (%)

1	2.5	2.5	3.5	90
2	2.0	2.0	4	90
3	1.5	1.5	6	75^a^
4	1.0	1.0	7	50^a^

^a^The rest of the starting material remained unreacted.

**Table 3:** Appel-reagent-mediated transformation of glycosyl hemiacetal derivatives to the thioglycosides at room temperature.

Entry	Glycosyl hemiacetal	Thioglycoside	Time (h)^a^	Yield (%)	Ref

1	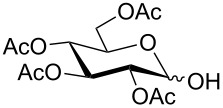 **1**	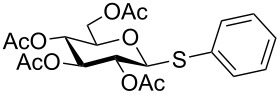 **10**	4	88	[49]
2	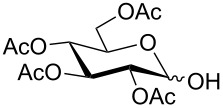 **1**	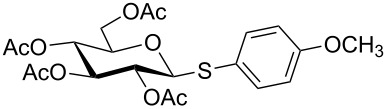 **11**	4	90	[50]
3	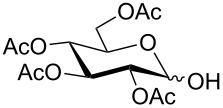 **1**	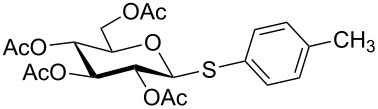 **12**	4	86	[51]
4	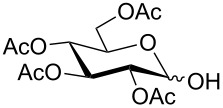 **1**	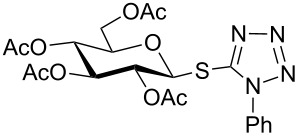 **13**	8	82	[52]
5	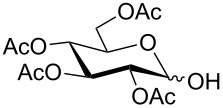 **1**	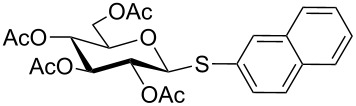 **14**	6	80	[23]
6	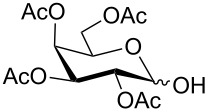 **2**	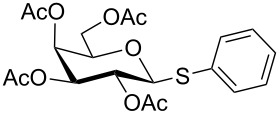 **15**	4	90	[53]
7	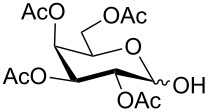 **2**	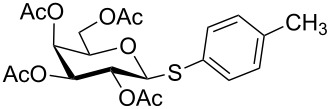 **16**	4	88	[51]
8	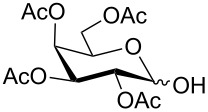 **2**	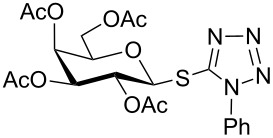 **17**	8	84	–
9	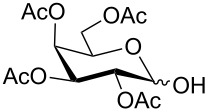 **2**	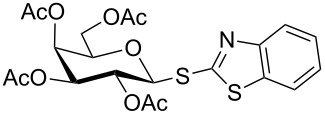 **18**	8	77	[54]
10	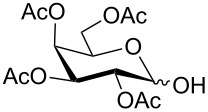 **2**	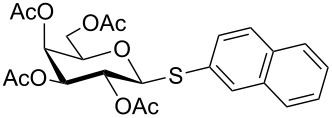 **19**	6	80	[23]
11	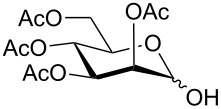 **3**	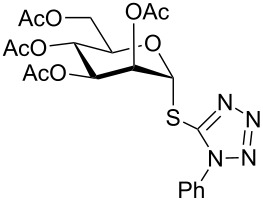 **20**	8	76	–
12	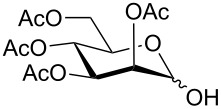 **3**	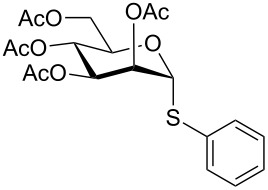 **21**	4	88	[53]
13	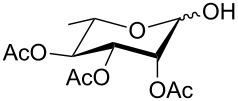 **4**	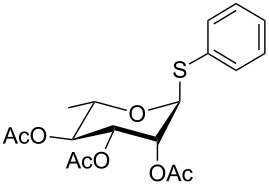 **22**	4	90	[55]
14	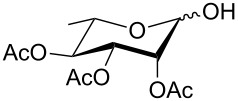 **4**	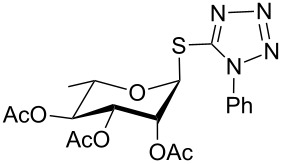 **23**	8	88	–
15	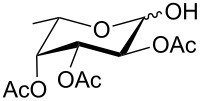 **5**	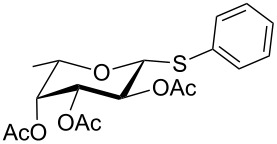 **24**	4	92	[56]
16	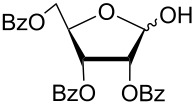 **6**	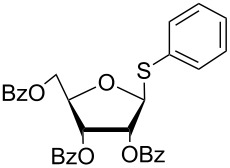 **25**	6	85	[57]
17	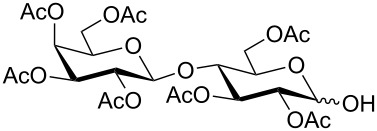 **7**	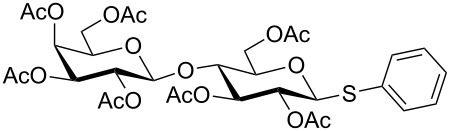 **26**	4	88	[25]
18	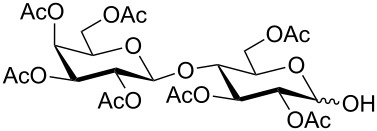 **7**	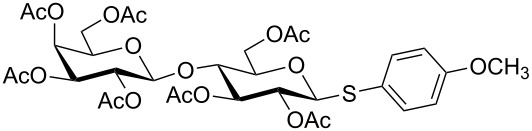 **27**	4	85	[50]
19	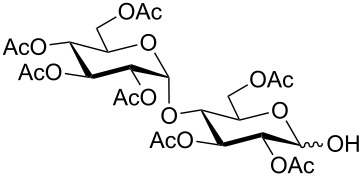 **8**	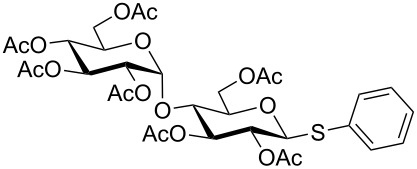 **28**	4	88	[25]
20	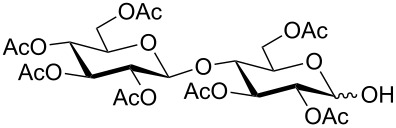 **9**	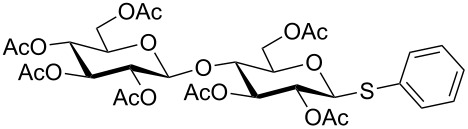 **29**	4	85	[25]

^a^Time for the second steps after the formation of glycosyl bromides in situ*.*

**Scheme 2:**
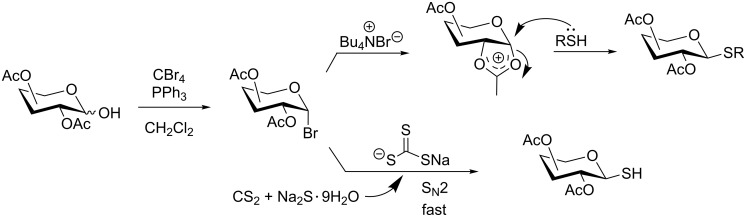
Plausible mechanistic pathways for the formation of 1,2-*trans*-thioglycoside and β-glycosyl thiol.

In another experiment, compound **1** (1.0 mmol) was treated with a mixture of CBr_4_ (2.0 mmol) and PPh_3_ (2.0 mmol) in CH_2_Cl_2_ (5 mL) at room temperature for 4 h. Addition of a red-colored solution of CS_2_ (2.0 mmol) and Na_2_S·9H_2_O (2.0 mmol) (sodium carbonotrithioate) in DMF (2 mL, prepared separately) to the reaction mixture furnished 2,3,4,6-tetra-*O*-acetyl-1-thio-β-D-glucopyranose (**30**) in 90% yield instantly. After optimization of the reaction condition it was observed that the best yield of compound **30** can be achieved by using CS_2_ (1.5 mmol) and Na_2_S·9H_2_O (2.0 mmol) at room temperature. On reduction of the quantity of CS_2_ and Na_2_S·9H_2_O, the rate of the reaction became very slow and a considerable amount of undesired byproducts were formed. The optimized reaction conditions were applied for the preparation of a series of glycosyl thiols in excellent yield in a stereoselective manner (Table 4). The reaction conditions have several notable features, which include (a) excellent yield; (b) exceptionally high stereoselectivity; (c) one-pot two-step reaction conditions; (d) applicability for scaled-up synthesis. It is worth mentioning that 1,2-*trans*-thioglycosides and exclusively β-glycosyl thiols were formed under these conditions. The reaction conditions have been applied successfully for the preparation of thioglycosides and glycosyl thiols from D- and L-sugars as well as disaccharides. The stereochemistry of the anomeric centers of the thioglycosides and glycosyl thiols were confirmed from their NMR spectral analysis (coupling constant of the H-1 (*J*_1,2_)). Appearance of the coupling constant of the H-1 (*J*_1,2_) = 8–10 Hz and coupling constant of the SH group (*J*_H-1,SH_) = 9–10 Hz in the ^1^H NMR spectra of the glycosyl thiols confirmed the exclusive formation of β-glycosyl thiols. In the case of D-mannose and L-rhamnose, exclusive formation of β-products were unambiguously confirmed from the coupling constant at the anomeric center (*J*_C-1,H-1_) in the gated ^1^H coupled ^13^C NMR spectra. Coupling constant (*J*_C-1,H-1_) = 143 Hz and 142 Hz (less than 160 Hz) in the gated ^1^H coupled ^13^C NMR spectra of compounds **32** and **33** confirmed the exclusive formation of β-products [47–48]. It is presumed that the reaction of the glycosyl hemiacetal derivative with the combination of CBr_4_ and PPh_3_ furnished α-glycosyl bromide in the first step. Interconversion of the α-glycosyl bromide to the reactive β-glycosyl bromide in the presence of a catalytic bromide ion derived from TBAB, led to the formation of a 1,2-oxocarbonium ion by participation of the neighboring group, which finally furnished 1,2-*trans*-thioglycoside by the reaction of thiols under reasonably slow biphasic reaction conditions. The thioglycoside formation became very slow without the addition of TBAB and the same product was obtained in a poor yield over a much longer period of time. In contrast, rapid S_N_2-substitution of the bromide ion at the anomeric center in α-glycosyl bromide with a carbonotrithioate ion (derived from the reaction of CS_2_ and Na_2_S·9H_2_O) in homogeneous solution led to the exclusive formation of the β-glycosyl thiol derivative (Scheme 2).

**Table 4:** Appel-reagent-mediated transformation of glycosyl hemiacetal derivatives to glycosyl thiol derivatives at room temperature.

Entry	Glycosyl hemiacetal	Glycosyl thiol	Time^a^ (min)	Yield (%)	Ref

1	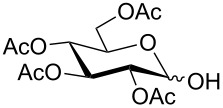 **1**	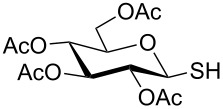 **30**	5	90	[41]
2	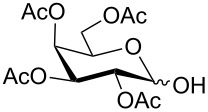 **2**	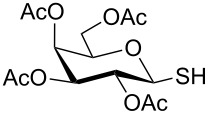 **31**	5	92	[58]
3	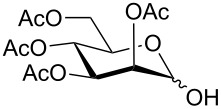 **3**	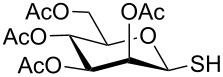 **32**	5	85	[59]
4	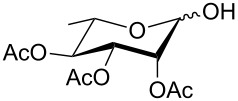 **4**	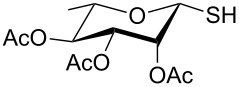 **33**	5	90	[60]
5	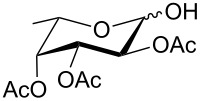 **5**	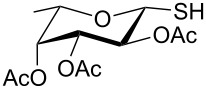 **34**	5	88	[41]
6	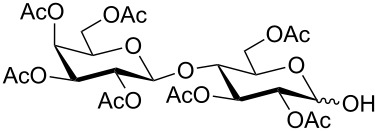 **7**	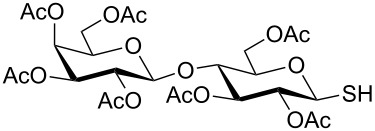 **35**	15	86	[61]
7	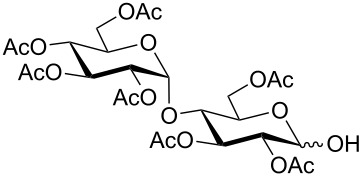 **8**	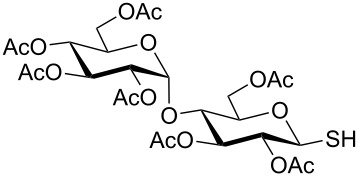 **36**	15	85	[62]

^a^Time for the second steps after formation of glycosyl bromides in situ.

## Conclusion

In summary, treatment of glycosyl hemiacetal derivatives with Appel reagent followed by reaction with thiols and sodium carbonotrithioate (derived from the reaction of CS_2_ and Na_2_S·9H_2_O) furnished thioglycosides and glycosyl thiols in excellent yield with high stereoselectivity in a two-step, one-pot reaction condition. The reaction condition is operationally simple, mild, reproducible, high-yielding, highly stereoselective, and can be scaled up for large-scale preparation. These reaction conditions may be considered as a valuable addition to those existing in this area.

## Experimental

**General methods:** All reactions were monitored by thin-layer chromatography over silica-gel-coated TLC plates. The spots on TLC were visualized by warming ceric sulfate (2% Ce(SO_4_)_2_ in 2 N H_2_SO_4_) sprayed plates on a hot plate. Silica gel 230–400 mesh was used for column chromatography. ^1^H and ^13^C NMR spectra were recorded on Bruker Avance 500 MHz by using CDCl_3_ as solvent and TMS as internal standard, unless stated otherwise. Chemical shift values are expressed as δ in parts per million. ESIMS were recorded on a Micromass mass spectrometer. Commercially available grades of organic solvents of adequate purity were used in all reactions.

### General experimental conditions for the preparation of thioglycosides

To a solution of glycosyl hemiacetal (1.0 mmol) in dry CH_2_Cl_2_ (5 mL) were added CBr_4_ (2.0 mmol) and PPh_3_ (2.0 mmol) and the reaction mixture was stirred at room temperature for 4 h. After consumption of the starting material (TLC, hexane–EtOAc 2:1), thiophenol (1.5 mmol), 10% aq. Na_2_CO_3_ (5 mL) and TBAB (20 mg) were added to the reaction mixture, and it was stirred for the appropriate time mentioned in Table 3. The reaction mixture was diluted with water and extracted with CH_2_Cl_2_ (50mL). The organic layer was washed with water, dried (Na_2_SO_4_) and concentrated to give the crude product, which was purified over SiO_2_ by using hexane–EtOAc as eluant to give the pure product. Known compounds gave spectral data identical to the data reported in the cited references.

**1-Phenyl-1*H*-tetrazol-5-yl 2,3,4,6-tetra-*O*-acetyl-1-thio-β-D-galactopyranoside (17)**: Yellow oil; [α]_D_^25^ +15 (*c* 1.2, CHCl_3_); IR (neat): 3114, 2842, 1612, 1522, 1467, 912, 699 cm^−1^; ^1^H NMR (500 MHz, CDCl_3_) δ 7.58–7.52 (m, 5H, Ar-H), 5.80 (d, *J* = 10.0 Hz, 1H, H-1), 5.46 (d, *J* = 3.0 Hz, 1H, H-4), 5.34 (t, *J* = 10.0 Hz each, 1H, H-2), 5.17 (dd, *J* = 10.0, 3.5 Hz, 1H, H-3), 4.12–4.10 (m, 2H, H-5, H-6_a_), 4.08–4.06 (m, 1H, H-6_b_), 2.15, 2.05, 2.01, 1.99 (4 s, 12H, 4 COC*H*_3_); ^13^C NMR (125 MHz, CDCl_3_) δ 170.1, 169.9, 169.6, 169.5 (4 *C*OCH_3_), 133.3–124.1 (Ar-C), 84.2 (C-1), 75.2 (C-3), 71.6 (C-4), 67.1 (C-5), 67.0 (C-2), 60.9 (C-6), 20.7, 20.6, 20.5, 20.4 (4 CO*C*H_3_); ESIMS (*m*/*z*): 531.1 [M + Na]^+^; Anal. calcd for C_21_H_24_N_4_O_9_S (508.12): C, 49.60; H, 4.76; found: C, 49.45; H, 4.94.

**1-Phenyl-1*H*-tetrazol-5-yl 2,3,4,6-tetra-*O*-acetyl-1-thio-α-D-mannopyranoside (20)**: Yellow oil; [α]_D_^25^ −2 (*c* 1.2, CHCl_3_); IR (neat): 3104, 2838, 1610, 1520, 1472, 916, 697 cm^−1^; ^1^H NMR (500 MHz, CDCl_3_) δ 7.61–7.50 (m, 5H, Ar-H), 6.12 (br s, 1H, H-1), 5.68 (d, *J* = 2.5 Hz, 1H, H-2), 5.27 (t, *J* = 10.0 Hz each, 1H, H-4), 5.19 (dd, *J* = 10.0, 3.0 Hz, 1H, H-3), 4.31 (dd, *J* = 12.5, 5.5 Hz, 1H, H-6_a_), 4.13 (d, *J* = 12.5 Hz, 1H, H-6_b_), 3.91–3.88 (m, 1H, H-5), 2.19, 2.07, 2.05, 1.99 (4 s, 12H, 4 COC*H*_3_); ^13^C NMR (125 MHz, CDCl_3_) δ 170.1, 170.0, 169.7, 169.6 (4 *C*OCH_3_), 130.5–124.1 (Ar-C), 82.6 (C-1), 77.2 (C-3), 71.5 (C-4), 70.1 (C-5), 65.0 (C-2), 62.0 (C-6), 20.7, 20.6, 20.5, 20.4 (4 CO*C*H_3_); ESIMS (*m*/*z*): 531.1 [M + Na]^+^; Anal. calcd for C_21_H_24_N_4_O_9_S (508.12): C, 49.60; H, 4.76; found: C, 49.42; H, 4.97.

**1-Phenyl-1*H*-tetrazol-5-yl 2,3,4-tri-*O*-acetyl-1-thio-α-L-rhamnopyranoside (23)**: Yellow oil; [α]_D_^25^ +19 (*c* 1.2, CHCl_3_); IR (neat): 3106, 2836, 1600, 1502, 1457, 916, 699 cm^−1^; ^1^H NMR (500 MHz, CDCl_3_) δ 7.59–7.50 (m, 5H, Ar-H), 6.10 (d, *J* = 1.0 Hz, 1H, H-1), 5.66 (d, *J* = 3.5, 1.0 Hz, 1H, H-2), 5.13 (dd, *J* = 10.0, 3.0 Hz, 1H, H-3), 5.06 (t, *J* = 9.5 Hz each, 1H, H-4), 3.80–3.75 (m, 1H, H-5), 2.18, 2.07, 1.97 (3 s, 9H, 3 COC*H*_3_), 1.28 (d, *J* = 6.0 Hz, 3H, C*H*_3_); ^13^C NMR (125 MHz, CDCl_3_) δ 170.1, 170.0, 169.9 (3 *C*OCH_3_), 130.4–124.1 (Ar-C), 82.4 (C-1), 75.6 (C-5), 71.4 (C-3), 70.5 (C-4), 69.8 (C-2), 20.7, 20.6, 20.5 (3 CO*C*H_3_), 17.6 (*C*H_3_); ESIMS (*m*/*z*): 473.1 [M + Na]^+^; Anal. calcd for C_19_H_22_N_4_O_7_S (450.12): C, 50.66; H, 4.92; found: C, 50.47; H, 5.15.

### General experimental condition for the preparation of glycosyl thiol derivatives

To a solution of glycosyl hemiacetal (1.0 mmol) in dry CH_2_Cl_2_ (5 mL) were added CBr_4_ (2.0 mmol) and PPh_3_ (2.0 mmol), and the reaction mixture was stirred for 4 h at room temperature. After completion of the reaction (TLC; hexane–EtOAc 2:1), a red premixed solution of CS_2_ (1.5 mmol) and Na_2_S·9H_2_O (2.0 mmol) in DMF (2 mL) was added to the reaction mixture and it was stirred for the appropriate time mentioned in Table 4. The reaction mixture was diluted with water and extracted with CH_2_Cl_2_ (50 mL). The organic layer was washed with water, dried (Na_2_SO_4_) and concentrated to give the crude product, which was purified over SiO_2_ by using hexane–EtOAc as eluant to give the pure product. Known compounds gave spectral data identical to the data reported in the cited references.

## Supporting Information

File 1 Analytical data of compounds **10**–**16**, **18**, **19**, **21**, **22**, **24-36** and ^1^H NMR, ^13^C NMR spectra of compounds **17**, **20**, and **23**.
