# Supplementary material for: Appel-reagent-mediated transformation of glycosyl hemiacetal derivatives into thioglycosides and glycosyl thiols
Source: Beilstein J Org Chem. 2013 May 22;9:974–82. doi: 10.3762/bjoc.9.112 (PMC3678522; doi:10.3762/bjoc.9.112)
Supplement: File 1 — Analytical data of compounds 10–16, 18, 19, 21, 22, 24-36 and 1H NMR, 13C NMR spectra of compounds 17, 20, and 23. [file Beilstein_J_Org_Chem-09-974-s001.pdf]

Supporting Information

for

Appel-reagent-mediated transformation of glycosyl hemiacetal derivatives into thioglycosides and glycosyl thiols

Tamashree Ghosh[‡], Abhishek Santra[‡] and Anup Kumar Misra[*]

Address: Bose Institute, Division of Molecular Medicine, P-1/12, C.I.T. Scheme VII-M, Kolkata-700054, India, FAX: 91-33-2355 3886

Email: Anup Kumar Misra - akmisra69@gmail.com

* Corresponding author

[‡]equally contributing authors

Analytical data of compounds 10–16, 18, 19, 21, 22, 24-36 and ^{1}H NMR, ^{13}C NMR spectra of compounds 17, 20, and 23.

Table of Contents

Spectral data of compounds **10–16, 18, 19, 21, 22, 24–36** S2–S9

Copies of the NMR spectra of compounds **17**, **20** and **23** S10–S12

Spectral data of synthesized compounds:

Phenyl 2,3,4,6-tetra-*O*-acetyl-1-thio-β-D-glucopyranoside (10)[1]: White solid; mp 117–118 °C; $[\alpha]_D^{25}$ −15 (*c* 1.2, $CHCl_3$); IR (KBr): 3024, 1750, 1477, 1436, 1371, 1237, 1067, 917, 763 cm $^{-1}$; ^{1}H NMR ($CDCl_3$, 300 MHz): δ 7.51-7.29 (m, 5 H, Ar-H), 5.22-5.13 (t, *J* = 9.2 Hz each, 1 H, H-3), 5.08-5.02 (t, *J* = 9.7 Hz each, 1 H, H-2), 4.98-4.92 (t, *J* = 9.2 Hz each, 1 H, H-4), 4.70 (d, *J* = 9.9 Hz, 1 H, H-1), 4.21-4.18 (m, 2 H, H-$6_{a,b}$), 3.80-3.65 (m, 1 H, H-5), 2.08, 2.07, 2.01, 1.98 (4 s, 12 H, 4 $COCH_3$); ^{13}C NMR ($CDCl_3$, 75 MHz): δ 170.3, 170.1, 169.2, 169.0, 133.7-128.7 (Ar-C), 85.9, 76.2, 74.4, 70.3, 68.5, 62.3, 20.9 (2 C), 20.8 (2 C); ESIMS: 463.1 $[M + Na]^+$; Anal. calcd for $C_{20}H_{24}O_9S$ (440.11): C, 54.54; H, 5.49; found: C, 54.40; H, 5.70.

4-Methoxyphenyl 2,3,4,6-tetra-*O*-acetyl-1-thio-β-D-glucopyranoside (11)[2]: White solid; mp 164–165 °C; $[\alpha]_D^{25}$ −22 (*c* 1.2, $CHCl_3$); ^{1}H NMR ($CDCl_3$, 500 MHz): δ 7.44 (d, *J* = 9.2 Hz, 2 H, Ar-H), 6.84 (d, *J* = 9.2 Hz, 2 H, Ar-H), 5.19 (t, *J* = 9.2 Hz each, 1 H, H-3), 4.99 (t, *J* = 9.8 Hz each, 1 H, H-2), 4.89 (t, *J* = 9.2 Hz each, 1 H, H-4), 4.54 (d, *J* = 9.8 Hz, 1 H, H-1), 4.22-4.15 (m, 2 H, H-6), 3.81 (s, 3 H, OCH_3), 3.69-3.65 (m, 1 H, H-5), 2.10, 2.07, 2.01, 1.98 (4 s, 12 H, 4 $COCH_3$); ^{13}C NMR ($CDCl_3$, 125 MHz): δ 170.6, 170.2, 169.4, 169.2 (4 $COCH_3$), 160.4-114.4 (Ar-C), 85.6 (C-1), 75.7, 74.0, 69.8, 68.2, 62.0 (C-6), 55.3 (OCH_3), 20.8, 20.7 (2 C), 20.6 (4 $COCH_3$); ESIMS: 493.1 $[M + Na]^+$; Anal. calcd for $C_{21}H_{26}O_{10}S$ (470.12): C, 53.61; H, 5.57; found: C, 53.42; H, 5.64.

***p*-Methylphenyl 2,3,4,6-tetra-*O*-acetyl-1-thio-β-D-glucopyranoside (12)[3]:** White solid; mp 117–118 °C; $[\alpha]_D^{25}$ −22 (*c* 1.2, $CHCl_3$); IR (KBr): 2925, 2363, 1749, 1221, 771 cm $^{-1}$; ^{1}H NMR ($CDCl_3$, 300 MHz): δ 7.41-7.37 (m, 2 H, Ar-H), 7.14-7.10 (m, 2 H, Ar-H), 5.25-5.16 (t, *J* = 9.2 Hz each, 1 H), 5.07-4.97 (t, *J* = 9.8, 9.5 Hz, 1 H), 4.98-4.88 (t, *J* = 9.8 Hz each, 1 H), 4.63 (d, *J* = 9.9 Hz, 1 H), 4.21-4.13 (m, 2 H, H-$6_{a,b}$), 3.74-3.65 (m, 1 H, H-5), 2.35, 2.09, 2.07, 2.01, 1.98 (5 s, 15 H); ^{13}C NMR ($CDCl_3$, 50 MHz): δ 170.9, 170.5, 169.7, 169.6, 139.1-127.9 (Ar-C), 86.2, 76.1, 74.4, 70.3, 68.6, 62.5, 21.6, 21.2, 21.1, 20.9 (2 C); ESIMS: 477.1 $[M + Na]^+$; Anal. calcd for $C_{21}H_{26}O_9S$ (454.12): C, 55.50; H, 5.77; found: C, 55.33; H, 5.62.

1-Phenyl-1*H*-tetrazol-5-yl 2,3,4,6-tetra-*O*-acetyl-β-D-glucopyranoside (13)[4]: Yellow oil; $[\alpha]_D^{25}$ −8 (*c* 1.2, $CHCl_3$); IR (neat): 3110, 2840, 1610, 1520, 1460, 910, 696 cm $^{-1}$; ^{1}H NMR (500 MHz, $CDCl_3$): δ 7.58-7.52 (m, 5 H, Ar-H), 5.78 (d, *J* = 10.0 Hz, 1 H, H-1), 5.31 (t, *J* = 9.0 Hz each, 1 H, H-3), 5.18 (t, *J* = 10.0 Hz each, 1 H, H-2), 5.12 (t, *J* = 10.0 Hz each, 1 H, H-4), 4.29-4.07 (m, 2 H, H-6_{ab}), 3.92-3.89 (m, 1 H, H-5), 2.06, 2.05, 2.04, 2.01 (4 s, 12 H, 4 $COCH_3$); ^{13}C NMR (125 MHz, $CDCl_3$): δ 170.3, 169.7, 169.3, 169.2 (4 $COCH_3$), 133.3-124.0 (Ar-C), 83.7 (C-1), 76.5 (C-5), 73.6

(C-3), 69.8 (C-4), 67.7 (C-2), 61.3 (C-6), 20.7, 20.6, 20.5, 20.4 (4 CO*C*H_3); ESIMS: 531.1 $[M + Na]^+$; Anal. calcd for $C_{21}H_{24}N_4O_9S$ (508.12): C, 49.60; H, 4.76; found: C, 49.45; H, 4.90.

2-Naphthyl 2,3,4,6-tetra-*O*-acetyl-1-thio-β-D-glucopyranoside (14)[5]: White solid; mp 110–112 °C; $[\alpha]_D$ −18 (*c* 1.2, $CHCl_3$); IR (KBr): 1747, 1594, 1381, 1225, 1045 cm $^{-1}$; ^{1}H NMR ($CDCl_3$, 200 MHz): δ 7.97 (bs, 1 H), 7.82-7.73 (m, 3 H), 7.56-7.45 (m, 3 H), 5.19 (t, *J* = 9.1 Hz each, 1 H, H-2), 4.99 (t, *J* = 9.6 Hz each, 1 H, H-3), 4.96 (t, *J* = 9.5 Hz each, 1 H, H-4), 4.73 (d, *J* = 9.9 Hz, 1 H, H-1), 4.21-4.10 (m, 2 H, H-$6_{a,b}$), 3.73-3.65 (m, 1 H, H-5), 2.10, 2.02, 2.0, 1.97 (4 s, 12 H, 4 $COCH_3$); ^{13}C NMR ($CDCl_3$, 50 MHz): δ 170.4, 170.1, 169.3, 169.1, 133.8, 133.3, 130.8, 129.0, 128.7, 128.1 (2 C), 127.0 (2 C), 126.9, 85.9, 76.3, 74.3, 70.4, 68.5, 62.3, 21.0, 20.9, 20.8 (2 C); ESIMS: 513.1 $[M + Na]^+$; Anal. calcd for $C_{24}H_{26}O_9S$ (490.12): C, 58.77; H, 5.34; found: C, 58.60; H, 5.50.

Phenyl 2,3,4,6-tetra-*O*-acetyl-1-thio-β-D-galactopyranoside (15)[6]: Yellow oil; $[\alpha]_D^{25}$ +3 (*c* 1.5, $CHCl_3$); IR (neat): 2363, 1594, 1353, 1051 cm $^{-1}$; ^{1}H NMR ($CDCl_3$, 300 MHz): δ 7.54-7.29 (m, 5 H, Ar-H), 5.42 (d, *J* = 3.1 Hz, 1 H, H-4), 5.29-5.19 (t, *J* = 10.7, 9.8 Hz, 1 H, H-2), 5.09-5.02 (dd, *J* = 9.9, 3.2 Hz, 1 H, H-3), 4.75 (d, *J* = 9.8 Hz, 1 H, H-1), 4.18-4.10 (m, 2 H, H-$6_{a,b}$), 3.98-391 (m, 1 H, H-5), 2.11, 2.09, 2.03, 1.97 (4 s, 12 H, 4 COC*H_3*); ^{13}C NMR ($CDCl_3$, 75 MHz): δ 170.7, 170.5, 170.3, 169.7, 132.9-128.5 (Ar-C), 86.9, 74.8, 72.4, 67.7 (2 C), 62.0, 21.2 (2 C), 20.9 (2 C); ESIMS: 463.1 $[M + Na]^+$; Anal. calcd for $C_{20}H_{24}O_9S$ (440.11): C, 54.54; H, 5.49; found: C, 54.38; H, 5.70.

***p*-Methylphenyl 2,3,4,6-tetra-*O*-acetyl-1-thio-β-D-galactopyranoside (16)[3]:** Yellow oil; $[\alpha]_D^{25}$ −5 (*c* 1.0, $CHCl_3$); IR (neat): 2928, 2367, 1741, 1597, 1353, 1227, 1048, 768 cm $^{-1}$; ^{1}H NMR ($CDCl_3$, 300 MHz): δ 7.43-7.39 (d, *J* = 8.1 Hz, 2 H, Ar-H), 7.14-7.10 (d, *J* = 8.0 Hz, 2 H, Ar-H), 5.41-5.39 (d, *J* = 3.1 Hz, 1 H, H-4), 5.27-5.17 (t, *J* = 9.8 Hz each, 1 H, H-2), 5.06-5.00 (dd, *J* = 9.8, 3.2 Hz, 1 H, H-3), 4.67-4.62 (d, *J* = 9.8 Hz, 1 H, H-1), 4.24-4.06 (m, 2 H, H-6_{ab}), 3.94-3.88 (t, *J* = 6.5 Hz each, 1 H, H-5), 2.34 (s, 3 H, PhC*H_3*), 2.11, 2.10, 2.04, 1.97 (4 s, 12 H, 4 COC*H_3*); ^{13}C NMR ($CDCl_3$, 50 MHz): δ 170.7, 170.6, 170.4, 169.8, 138.8-129.0 (Ar-C), 87.3, 74.7, 72.4, 67.7, 67.6, 61.9, 21.5 21.2, 20.9 (3 C); ESIMS: 477.1 $[M + Na]^+$; Anal. calcd for $C_{21}H_{26}O_9S$ (454.12): C, 55.50; H, 5.77; found: C, 55.35; H, 5.60.

2-Benzothiazolyl 2,3,4,6-tetra-*O*-acetyl-1-thio-β-D-galactopyranoside (18)[7]: Yellow oil; $[\alpha]_D^{25}$ +34 (*c* 1.0, $CHCl_3$); IR (neat): 2908, 2377, 1751, 1600, 1343, 1236, 1054, 767 cm $^{-1}$; ^{1}H NMR ($CDCl_3$, 300 MHz): δ 7.94-7.33 (m, 4 H, Ar-H), 5.57 (d, *J* = 10.5 Hz, 1 H, H-1), 5.49 (d, *J* = 3.5 Hz, 1 H, H-4), 5.40 (t, *J* = 10.0 Hz, 1 H, H-2), 5.17 (dd, *J* = 10.0, 3.5 Hz, 1 H, H-3), 4.18-4.10 (m, 3 H, H-5, H-6_{ab}), 2.18, 2.04, 2.00, 1.99 (4 s, 12 H, 4 COC*H_3*); ^{13}C NMR (125 MHz, $CDCl_3$): δ 170.3, 170.1, 170.0, 169.9 (4 *C*OCH_3), 126.3-120.9 (Ar-C), 84.5 (C-1), 74.9 (C-3), 71.8 (C-4), 67.1 (C-2),

66.9 (C-5), 61.2 (C-6), 20.7, 20.6 (2 C), 20.5 (4 CO*C*H_3); ESIMS: 520.1 $[M + Na]^+$; Anal. calcd for $C_{21}H_{23}NO_9S_2$ (497.08): C, 50.69; H, 4.66; found: C, 50.53; H, 4.80.

2-Naphthyl 2,3,4,6-tetra-*O*-acetyl-1-thio-β-D-galactopyranoside (19)[5]: White solid; mp 114–116 °C; $[\alpha]_D$ +5 (*c* 1.2, $CHCl_3$); IR (KBr): 1743, 1592, 1375, 1224, 1043 cm $^{-1}$; ^{1}H NMR ($CDCl_3$, 200 MHz): δ 7.99 (bs, 1 H), 7.82-7.74 (m, 3 H), 7.57-7.44 (m, 3 H), 5.36 (d, *J* = 2.8 Hz, 1 H, H-4), 5.20 (t, *J* = 9.8 Hz each, 1 H, H-2), 5.02 (dd, *J* = 9.6, 3.2 Hz, 1 H, H-3), 4.75 (d, *J* = 9.7 Hz, 1 H, H-1), 4.19-4.06 (m, 2 H, H-$6_{a,b}$), 3.92-3.89 (m, 1 H, H-5), 2.11, 2.03, 2.0, 1.96 (4 s, 12 H, 4 $COCH_3$); ^{13}C NMR ($CDCl_3$, 50 MHz): δ 170.7, 170.5, 170.4, 169.8, 133.8, 133.1, 132.3, 130.1, 129.9, 128.8, 128.1, 128.0, 127.0 (2 C), 86.9, 74.9, 72.4, 67.7, 67.6, 62.1, 21.2, 21.0, 20.9 (2 C); ESIMS: 513.1 $[M + Na]^+$; Anal. calcd for $C_{24}H_{26}O_9S$ (490.12): C, 58.77; H, 5.34; found: C, 58.58; H, 5.52.

Phenyl 2,3,4,6-tetra-*O*-acetyl-1-thio-α-D-mannopyranoside (21)[6]: Yellow oil; $[\alpha]_D^{25}$ +118 (*c* 1.5, $CHCl_3$); IR (neat): 2930, 2121, 1753, 1370, 1220, 1052, 769 cm $^{-1}$; ^{1}H NMR ($CDCl_3$, 300 MHz): δ 7.51-7.46 (m, 2 H, Ar-H), 7.32-7.26 (m, 3 H, Ar-H), 5.49 (bs, 2 H, H-1 and H-2), 5.39-5.26 (m, 2 H, H-3, H-4), 4.62-4.44 (m, 1 H, H-5), 4.35-4.06 (ddd, *J* = 12.1, 2.16, 2.24 Hz, 2 H, H-$6_{a,b}$), 2.15, 2.07, 2.05, 2.02 (4 s, 12 H, 4 COC*H_3*); ^{13}C NMR ($CDCl_3$, 50 MHz): δ 170.9, 170.5, 170.1 (2 C), 133.0-128.5 (Ar-C), 86.0, 71.3, 69.9 (2 C), 66.7, 62.8, 21.3, 21.1, 21.0 (2 C); ESIMS: 463.1 $[M + Na]^+$; Anal. calcd for $C_{20}H_{24}O_9S$ (440.11): C, 54.54; H, 5.49; found: C, 54.40; H, 5.70.

Phenyl 2,3,4-tri-*O*-acetyl-1-thio-α-L-rhamnopyranoside (22)[8]: Yellow oil; $[\alpha]_D^{25}$ +76 (*c* 1.5, $CHCl_3$); IR (neat): 2354, 1763, 1220, 1067, 770 cm $^{-1}$; ^{1}H NMR ($CDCl_3$, 300 MHz): δ 7.52-7.28 (m, 5 H, Ar-H), 5.66 (d, *J* = 2.8 Hz, 1 H), 5.13-5.03 (t, *J* = 9.3 Hz each, 1 H), 5.03-4.98 (dd, *J* = 8.0, 3.3 Hz, 1 H), 4.90 (br s, 1 H), 3.59-3.51 (m, 1 H), 2.19, 2.04, 1.97 (3 s, 9 H, 3 COC*H_3*), 1.30 (d, *J* = 6.1 Hz, 3 H); ^{13}C NMR ($CDCl_3$, 75 MHz): δ 170.5, 170.4, 170.1, 133.7-128.2 (Ar-C), 85.7, 75.3, 72.2, 71.4, 70.6, 21.1, 20.9 (2 C), 18.1; ESIMS: 405.1 $[M + Na]^+$; Anal. calcd for $C_{18}H_{22}O_7S$ (382.10): C, 56.53; H, 5.80; found: C, 56.35; H, 6.00.

Phenyl 2,3,4-tri-*O*-acetyl-1-thio-β-L-fucopyranoside (24)[9]: Yellow oil; $[\alpha]_D^{25}$ –8 (*c* 1.5, $CHCl_3$); IR (neat): 2360, 1767, 1232, 1066, 772 cm^{-1}; ^{1}H NMR ($CDCl_3$, 300 MHz): δ 7.53-7.29 (m, 5 H, Ar-H), 5.27 (br s, 1 H, H-4), 5.22 (t, *J* = 9.8 Hz each, 1 H, H-2), 5.06 (dd, *J* = 9.8, 3.2 Hz, 1 H, H-3), 4.72 (d, *J* = 9.8 Hz, 1 H, H-2), 3.87-3.81 (m, 1 H, H-5), 2.14, 2.07, 1.96 (3 s, 9 H, 3 COC*H_3*), 1.25 (d, *J* = 6.0 Hz, 3 H, CC*H_3*); ^{13}C NMR (75 MHz): δ 170.9, 170.5, 169.8 (3 *C*OCH_3), 133.2-128.3 (Ar-C), 86.7 (C-1), 73.5 (C-3), 72.8 (C-4), 70.7 (C-2), 67.8 (C-5), 21.2, 20.9 (2 C) (3 CO*C*H_3), 16.8 (C*C*H_3); ESIMS: 405.1 $[M + Na]^+$; Anal. calcd for $C_{18}H_{22}O_7S$ (382.10): C, 56.53; H, 5.80; found: C, 56.37; H, 6.00.

Phenyl 2,3,5-tri-*O*-benzoyl-1-thio-β-D-ribofuranoside (25)[10]: Yellow oil; $[\alpha]_D^{25}$ +16 (*c* 1.5, $CHCl_3$); IR (neat): 2324, 1760, 1200, 1069, 776 cm^{-1}; ^1H NMR ($CDCl_3$, 300 MHz): δ 8.06-7.32 (m, 20 H, Ar-H), 5.73 (t, *J* = 5.0 Hz each, 1 H, H-2), 5.68 (t, *J* = 5.0 Hz each, 1 H, H-3), 5.62 (d, *J* = 4.9 Hz, 1 H, H-1), 4.68-4.62 (m, 2 H, H-4, H-5_a), 4.52 (dd, *J* = 11.5, 4.1 Hz, 1 H, H-5_b); ^{13}C NMR (125 MHz, $CDCl_3$): δ 166.6, 165.7, 165.5 (3 *C*OPh), 134.1-128.8 (Ar-C), 88.5 (C-1), 80.8 (C-3), 75.0 (C-2), 72.7 (C-4), 64.6 (C-5); ESIMS: 577.1 $[M + Na]^+$; Anal. calcd for $C_{32}H_{26}O_7S$ (554.14): C, 69.30; H, 4.73; found: C, 69.13; H, 4.95.

Phenyl 2,3,4,6-tetra-*O*-acetyl-β-D-galactopyranosyl-(1→4)-2,3,6-tri-*O*-acetyl-1-thio-β-D-glucopyranoside (26)[11]: White solid, mp 165–166 °C; $[\alpha]_D^{25}$ −19 (*c* 1.5, $CHCl_3$); IR (KBr): 2930, 1753, 1365, 1038, 752 cm^{-1}; ^1H NMR (300 MHz, $CDCl_3$): δ 7.47-7.44 (m, 2 H, Ar-H), 7.31-7.27 (m, 3 H, Ar-H), 5.32 (dd, *J* = 3.4 and 1.0 Hz, 1 H), 5.19 (dd, *J* = 9.2 and 9.1 Hz, 1 H), 5.08 (dd, *J* = 10.5 and 7.9 Hz, 1 H), 4.92 (t, *J* = 10.5 Hz each, 1 H), 4.88 (dd, *J* = 10.0 and 9.2 Hz, 1 H), 4.65 (d, *J* = 10.1 Hz, 1 H), 4.56 (dd, *J* = 11.9 and 2.0 Hz, 1 H), 4.44 (d, *J* = 7.9 Hz, 1 H), 4.10 (dd, *J* = 10.2 and 6.7 Hz, 1 H), 4.08 (dd, *J* = 11.9 and 5.6 Hz, 1H), 4.04 (dd, *J* = 10.2 and 7.2 Hz, 1 H), 3.86-3.83 (m, 1 H), 3.72 (dd, *J* = 9.9 and 9.1 Hz, 1 H), 3.67-3.60 (m, 1 H), 2.12, 2.08, 2.07, 2.02, 2.01, 2.00, 1.94 (7 s, 21 H, 7 COCH_3); ^{13}C NMR (75 MHz, $CDCl_3$): δ 170.3, 170.2, 170.1, 170.0, 169.7, 169.5, 169.0, 133.0-128.7 (Ar-C), 101.0, 85.5, 76.6, 76.1, 73.8, 70.9, 70.8, 70.2, 69.0, 66.6, 62.1, 60.8, 20.8 (2 C), 20.7 (2 C), 20.6 (2 C), 20.5; ESIMS: 751.1 $[M+Na]^+$; Anal. Calcd. for $C_{32}H_{40}O_{17}S$ (728.19): C, 52.74; H, 5.53; found: C, 52.55; H, 5.75.

4-Methoxyphenyl (2,3,4,6-tetra-*O*-acetyl-β-D-galactopyranosyl)-(1→4)-2,3,6-tri-*O*-acetyl-1-thio-β-D-glucopyranoside (27)[2]: White solid; mp 167–168 °C; $[\alpha]_D^{25}$ −21 (*c* 1.2, $CHCl_3$); IR (KBr): 2936, 1766, 1367, 1042, 756 cm^{-1}; ^1H NMR ($CDCl_3$, 500 MHz): δ 7.42 (d, *J* = 9.2 Hz, 2 H, Ar-H), 6.84 (d, *J* = 9.2 Hz, 2 H, Ar-H), 5.33 (br s, 1 H, H-4_B), 5.18 (t, *J* = 9.2 Hz each, 1 H, H-3_A), 5.08 (dd, *J* = 7.9, 7.9 Hz, 1 H, H-2_A), 4.94 (dd, *J* = 10.3, 3.4 Hz, 1 H, H-3_B), 4.80 (t, *J* = 9.7 Hz each, 1 H, H-2_B), 4.55-4.52 (dd, *J* = 11.9, 1.7 Hz, 1 H, H-6_{aA}), 4.50 (d, *J* = 10.0 Hz, 1 H, H-1_B), 4.44 (d, *J* = 7.9 Hz, 1 H, H-1_A), 4.13-4.0 (m, 3 H, H-6_{abB} and H-6_{bA}), 3.86-3.82 (m, 1 H, H-5_A), 3.80 (s, 3 H, OCH_3), 3.70 (t, *J* = 9.8 Hz each, 1 H, H-4_A), 3.59-3.56 (m, 1 H, H-5_B), 2.14, 2.10, 2.03, 2.02, 2.00, 1.95 (6 s, 21 H, 7 COCH_3); ^{13}C NMR ($CDCl_3$, 125 MHz): δ 170.3, 170.2, 170.1, 170.0, 169.7, 169.5, 169.0 (7 *C*OCH_3), 160.3-114.3 (Ar-C), 100.0 (C-1′), 85.4 (C-1), 76.6, 76.0, 73.9, 70.9, 70.6, 70.2, 69.0, 66.6, 61.9 (C-6), 60.7 (C-6′), 55.3 (O*C*H_3), 20.9, 20.8, 20.7, 20.6 (3 C), 20.5 (7 CO*C*H_3); ESIMS: 781.2 $[M + Na]^+$; Anal. calcd for $C_{33}H_{42}O_{18}S$ (758.20): C, 52.24; H, 5.58; found: C, 52.10; H, 5.75.

Phenyl 2,3,4,6-tetra-*O*-acetyl-α-D-glucopyranosyl-(1→4)-2,3,6-tri-*O*-acetyl-1-thio-β-D-glucopyranoside (28)[11]: White solid; mp 82–84 °C; $[\alpha]_D^{25}$ +47 (*c* 1.5, $CHCl_3$); IR (KBr): 2927, 1763, 1365, 1030, 756 cm^{-1}; ^1H NMR (300 MHz, $CDCl_3$): δ 7.48-7.45 (m, 2 H, Ar-H), 7.32-7.24 (m, 3 H, Ar-H), 5.39-5.29 (m, 3 H), 5.08 (t, *J* = 9.8 Hz each, 1 H), 4.88-4.75 (m, 3 H), 4.50 (d, *J* = 9.8 Hz, 1 H), 4.25-4.20 (m, 2 H), 4.07 (br s, 1 H), 4.01-3.88 (m, 2 H), 3.75-3.73 (m, 1 H), 2.08, 2.06, 2.05, 2.03, 2.02, 2.01, 1.98 (7 s, 21 H, 7 $COCH_3$); ^{13}C NMR (75 MHz, $CDCl_3$): δ 170.4, 170.2, 170.1 (2 C), 169.8, 169.4, 169.3, 133.3-128.4 (Ar-C), 95.5, 85.0, 76.4, 76.0, 72.4, 70.6, 69.9, 69.2, 68.5, 67.9, 62.7, 61.4, 20.8 (2 C), 20.7 (2 C), 20.6 (2 C), 20.5; ESIMS: 751.1 $[M + Na]^+$; Anal. calcd for $C_{32}H_{40}O_{17}S$ (728.19): C, 52.74; H, 5.53; found: C, 52.57; H, 5.72.

Phenyl 2,3,4,6-tetra-*O*-acetyl-β-D-glucopyranosyl-(1→4)-2,3,6-tri-*O*-acetyl-1-thio-β-D-glucopyranoside (29)[11]: White solid; mp 223–225 °C; $[\alpha]_D^{25}$ −29 (*c* 1.5, $CHCl_3$); IR (KBr): 2928, 1753, 1235, 1038, 760 cm^{-1}; ^1H NMR (300 MHz, $CDCl_3$): δ 7.46-7.43 (m, 2 H, Ar-H), 7.31-7.26 (m, 3 H, Ar-H), 5.18 (dd, *J* = 9.1 and 9.0 Hz, 1 H), 5.12 (dd, *J* = 9.4 and 9.1 Hz, 1 H), 5.04 (dd, *J* = 9.5 and 9.3 Hz, 1 H), 4.90 (dd, *J* = 9.2 and 8.0 Hz, 1 H), 4.89 (dd, *J* = 10.0 and 9.2 Hz, 1 H), 4.64 (d, *J* = 10.2 Hz, 1 H), 4.54 (dd, *J* = 11.9 and 1.9 Hz, 1 H), 4.47 (d, *J* = 7.8 Hz, 1 H), 4.36 (dd, *J* = 12.5 and 4.3 Hz, 1 H), 4.07 (dd, *J* = 11.9 and 5.4 Hz, 1 H), 4.00 (dd, *J* = 12.6 and 2.2 Hz, 1 H), 3.71 (dd, *J* = 10.0 and 9.0 Hz, 1 H), 3.65-3.60 (m, 2 H), 2.09, 2.06, 2.04, 2.00, 1.98, 1.96 (6 s, 21 H, 7 $COCH_3$); ^{13}C NMR (75 MHz, $CDCl_3$): δ 170.5, 170.3, 169.8, 169.6, 169.4 (2 C), 169.1, 133.0-128.3 (Ar-C), 100.7, 85.4, 76.6, 76.2, 73.4, 72.8, 71.8, 71.4, 70.0, 67.5, 61.8, 61.3, 20.6 (2 C), 20.5 (2 C), 20.4 (2 C), 20.3; ESIMS: 751.1 $[M + Na]^+$; Anal. calcd for $C_{32}H_{40}O_{17}S$ (728.19): C, 52.74; H, 5.53; found: C, 52.59; H, 5.70.

2,3,4,6-Tetra-*O*-acetyl-1-thio-β-D-glucopyranose (30)[12]: Yellow oil; $[\alpha]_D^{25}$ +13.3 (*c* 1.0, $CHCl_3$); ^1H NMR (500 MHz, $CDCl_3$): δ 5.15 (t, *J* = 9.5 Hz each, 1 H, H-2), 5.09 (t, *J* = 10 Hz each, 1 H, H-3), 4.96 (t, *J* = 9.5 Hz, 1 H, H-4), 4.52 (t, *J* = 9.0 Hz each, 1 H, H-1), 4.25 (dd, *J* = 12.5, 5.0 Hz, 1 H, H-6_a), 4.11 (dd, *J* = 12.5, 2.0 Hz, 1 H, H-6_b), 3.71-3.69 (m, 1 H, H-5), 2.28 (d, *J* = 10.0 Hz, 1 H, S*H*), 2.09, 2.07, 2.02, 2.01 (4 s, 12 H, 4 $COCH_3$); ^{13}C NMR (125 MHz, $CDCl_3$): δ 170.4, 169.9, 169.4, 169.1 (4 $COCH_3$), 78.7 (C-1), 76.1 (C-2), 73.6 (2 C, C-3, C-4), 68.1 (C-6), 20.7, 20.6 (2 C), 20.5 (4 $COCH_3$); ESIMS: 387.0 $[M + Na]^+$; Anal. calcd for $C_{14}H_{20}O_9S$ (364.08): C, 46.15; H, 5.53; found: C, 46.0; H, 5.70.

2,3,4,6-Tetra-*O*-acetyl-1-thio-β-D-galactopyranose (31)[13]: Yellow oil; $[\alpha]_D^{25}$ +104.7 (*c* 1.0, $CHCl_3$); ^1H NMR (500 MHz, $CDCl_3$): δ 5.39 (d, *J* = 3.0 Hz, 1 H, H-4), 5.13 (t, *J* = 9.5 Hz each, 1 H, H-2), 4.98 (dd, *J* = 10.5, 3.5 Hz, 1 H, H-3), 4.90 (t, *J* = 10.0 Hz each, 1 H, H-1), 4.11-4.09 (m, 2

H, H-6_{ab}), 3.93-3.90 (m, 1 H, H-5), 2.30 (d, *J* =10.0 Hz, 1 H, S*H*), 2.15, 2.07, 2.02, 1.96 (4 s, 12 H, 4 COCH_3); ^{13}C NMR (125 MHz, $CDCl_3$): δ 170.1, 170.0, 169.7, 169.6 (4 *C*OCH_3), 79.2 (C-1), 74.9 (C-2), 71.6 (C-3), 70.9 (C-4), 67.2 (C-5), 61.3 (C-6), 20.8, 20.6, 20.5 (2 C), (4 CO*C*H_3); ESIMS: 387.0 $[M + Na]^+$; Anal. calcd. for $C_{14}H_{20}O_9S$ (364.08): C, 46.15; H, 5.53; found: C, 46.0; H, 5.75.

2,3,4,6-Tetra-*O*-acetyl-1-thio-β-D-mannopyranose (32)[14]: White solid; mp 146 °C; $[\alpha]_D^{25}$ −4.9 (*c* 1.0, $CHCl_3$); ^{1}H NMR (500 MHz, $CDCl_3$): δ 5.41 (d, *J* = 3.5 Hz, 1 H, H-2), 5.19 (t, *J* = 10.0 Hz each, 1 H, H-4), 5.05 (dd, *J* = 10.0, 3.5 Hz, 1 H, H-3), 4.87 (d, *J* = 10.0 Hz, 1 H, H-1), 4.23 (dd, *J* = 12.5, 5.0 Hz, 1 H, H-6_a), 4.10 (dd, *J* = 12.5, 2.0 Hz, 1 H, H-6_b), 3.69-3.66 (m, 1 H, H-5), 2.50 (d, *J* = 10.0 Hz, 1 H, S*H*), 2.23, 2.09, 2.03, 1.97 (4 s, 12 H, 4 COCH_3); ^{13}C NMR (125 MHz, $CDCl_3$): δ 169.9, 169.8, 169.4 (2 C) (4 *C*OCH_3), 76.9 (C-1), 76.4 (C-2), 72.0 (C-3), 71.6 (C-4), 65.2 (C-5), 62.0 (C-6), 20.8, 20.7, 20.6, 20.5 (4 CO*C*H_3); ESIMS: 387.0 $[M + Na]^+$; Anal. calcd for $C_{14}H_{20}O_9S$ (364.08): C, 46.15; H, 5.53; found: C, 46.02; H, 5.72.

2,3,4-Tri-*O*-acetyl-1-thio-β-L-rhamnopyranose (33)[15]: Yellow oil; $[\alpha]_D^{25}$ +46.3 (*c* 1.0, $CHCl_3$); ^{1}H NMR (500 MHz, $CDCl_3$): δ 5.32 (br s, 1 H, H-2), 4.94-4.93 (m, 2 H, H-3, H-4), 4.78 (d, *J* = 10.0 Hz each, 1 H, H-1), 3.49-3.46 (m, 1 H, H-5), 2.39 (d, *J* = 10.0 Hz, 1 H, S*H*), 2.15, 1.97, 1.90 (3 s, 9 H, 3 COCH_3), 1.19 (d, *J* = 6.0 Hz, 3 H, CCH_3); ^{13}C NMR (125 MHz, $CDCl_3$): δ 170.0, 169.8, 169.7 (3 *C*OCH_3), 75.9 (C-1), 75.3 (C-3), 72.0 (C-4), 71.9 (C-2), 69.9 (C-5), 20.7, 20.6, 20.5 (CO*C*H_3), 17.7 (C*C*H_3); ESIMS: 329.0 $[M + Na]^+$; Anal. calcd for $C_{12}H_{18}O_7S$ (306.07): C, 47.05; H, 5.92; found: C, 46.90; H, 6.10.

2,3,4-Tri-*O*-acetyl-1-thio-β-L-fucopyranose (34)[12]: White solid; mp 108–110 °C; $[\alpha]_D^{25}$ −2.5 (*c* 1.0, $CHCl_3$); ^{1}H NMR (500 MHz, $CDCl_3$): δ 5.26 (d, *J* = 3.5 Hz, 1 H, H-4), 5.11 (t, *J* = 10.0 Hz each, 1 H, H-2), 4.98 (dd, *J* = 10.0, 3.5 Hz, 1 H, H-3), 4.46 (t, *J* = 10.0 Hz, 1 H, H-1), 3.85-3.79 (m, 1 H, H-5), 2.28 (d, *J* = 10.0 Hz, 1 H, S*H*), 2.18, 2.06, 1.96 (3 s, 9 H, 3 COCH_3), 1.21 (d, *J* = 6.0 Hz, 3 H, CCH_3); ^{13}C NMR (125 MHz, $CDCl_3$): δ 170.4, 169.8, 169.7 (3 *C*OCH_3), 78.9 (C-1), 73.8 (C-3), 72.0 (C-4), 71.0 (C-2), 70.3 (C-5), 20.8, 20.7, 20.6 (3 CO*C*H_3), 16.4 (C*C*H_3); ESIMS: 329.0 $[M + Na]^+$; Anal. calcd for $C_{12}H_{18}O_7S$ (306.07): C, 47.05; H, 5.92; found: C, 46.88; H, 6.14.

(2,3,4,6-Tetra-*O*-acetyl-β-D-galactopyranosyl)-(1→4)-2,3,6-tri-*O*-acetyl-1-thio-β-D-glucopyranose (35)[16]: Yellow oil; $[\alpha]_D^{25}$ +0.2 (*c* 1.0, $CHCl_3$); ^{1}H NMR (500 MHz, $CDCl_3$): δ 5.32 (d, *J* = 3.0 Hz, 1 H, H-4_B), 5.16 (t, *J* = 9.5 Hz each, 1 H, H-3_A), 5.07 (t, *J* = 8.0 Hz each, 1 H, H-2_A), 4.93 (dd, *J* = 10.5, 3.5 Hz, 1 H, H-3_B), 4.85 (t, *J* = 9.5 Hz each, 1 H, H-2_B), 4.50 (t, *J* = 9.5 Hz each, 1 H, H-1_A), 4.45 (d, *J* = 8.0 Hz, 1 H, H-1_B), 4.42 (dd, *J* = 12.0,1.5 Hz, 1 H, H-6_{aB}), 4.13-4.05 (m, 3 H, H-6_{abA}, H-6_{aB}), 3.87-3.84 (m, 1 H, H-5_B), 3.78 (t, *J* = 10.0 Hz each, 1 H, H-4_A), 3.63-3.61 (m, 1

H, H-5_A), 2.23 (d, *J* = 10.0 Hz, 1 H, S*H*), 2.15, 2.13, 2.07, 2.06, 2.04, 1.96 (6 s, 21 H, 7 COCH_3); ^{13}C NMR (125 MHz, $CDCl_3$): δ 170.2, 170.1, 169.9, 169.8, 169.7, 169.4, 168.9 (7 *C*OCH_3), 101.1 (C-1_B), 78.5 (C-1_A), 77.1 (C-5_A), 76.1 (C-4_A), 73.9 (C-3_A), 73.5 (C-2_B), 70.9 (C-3_B), 70.7 (C-5_B), 69.0 (C-2_A), 66.5 (C-4_B), 62.2 (C-6_B), 60.7 (C-6_A), 20.8, 20.7 (2 C), 20.6 (2 C), 20.5, 20.4 (7 CO*C*H_3); ESIMS: 675.1 $[M + Na]^+$; Anal. calcd for $C_{26}H_{36}O_{17}S$ (652.16): C, 47.85; H, 5.56; found: C, 47.70; H, 5.74.

(2,3,4,6-Tetra-*O*-acetyl-α-D-glucopyranosyl-(1→4)-2,3,6-tri-*O*-acetyl-1-thio-β-D-glucopyranoside (36)[17]: White solid; mp 156–157 °C; $[\alpha]_D^{25}$ +70.7 (*c* 1.0, $CHCl_3$); ^{1}H NMR (500 MHz, $CDCl_3$): δ 5.32 (d, *J* = 4.0 Hz, 1 H, H-1_B), 5.25 (t, *J* = 10.0 Hz each, 1 H, H-3_A), 5.15 (t, *J* = 9.0 Hz each, 1 H, H-2_A), 4.95 (t, *J* = 10.0 Hz each, 1 H, H-3_B), 4.76 (dd, *J* = 10.5, 4.0 Hz, 1 H, H-2_B), 4.69 (t, *J* = 9.5 Hz each, 1 H, H-4_B), 4.50 (t, *J* = 9.5 Hz each, 1 H, H-1_A), 4.35 (dd, *J* = 12.0, 2.5 Hz, 1 H, H-6_{aA}), 4.16-4.12 (m, 2 H, H-6_{abB}), 3.97 (dd, *J* = 12.0, 1.5 Hz, 1 H, H-6_{bA}), 3.92 (t, *J* = 10.0 Hz each, 1 H, H-4_A), 3.88-3.84 (m, 1 H, H-5_A), 3.63-3.60 (m, 1 H, H-5_B), 2.15 (d, *J* = 10.0 Hz, 1 H, S*H*), 2.08, 2.02, 1.97, 1.96, 1.94, 1.93, 1.92 (7 s, 21 H, 7 COCH_3); ^{13}C NMR (125 MHz, $CDCl_3$): δ 170.4, 170.3, 170.2, 169.9, 169.7, 169.6, 169.2 (7 *C*OCH_3), 95.6 (C-1_B), 78.2 (C-1_A), 76.6 (C-2_A), 76.1 (C-5_B), 74.4 (C-4_B), 72.6 (C-4_A), 70.0 (C-2_B), 69.3 (C-3_A), 68.6 (C-3_B), 68.0 (C-5_A), 63.0 (C-6_A), 61.4 (C-6_B), 20.8, 20.7 (2 C), 20.6, 20.5 (3C) (7 COCH_3); ESIMS: 675.1 $[M + Na]^+$; Anal. calcd for $C_{26}H_{36}O_{17}S$ (652.16): C, 47.85; H, 5.56; found: C, 47.68; H, 5.75.

References

1. Dasgupta, F.; Garegg, P. J. *Acta Chem. Scand.* **1989**, *43*, 471-475.
2. Santra, A.; Sau, A.; Misra, A. K. *J. Carbohydr. Chem.* **2011**, *30*, 85-93.
3. Tai, C. A.; Kulkarni, S. S.; Hung, S. C. *J. Org. Chem.* **2003**, *68*, 8719-8722.
4. Couri, M. R.; Luduvico, I.; Santos, L.; Alves, R.; Prado, M. A.; Gil, R. F. *Carbohydr. Res.* **2007**, *342*, 1096-1100.
5. Mukherjee, C.; Misra, A. K. *J. Carbohydr. Chem.* **2007**, *26*, 213-221.
6. Khiar, N.; Martin-Lomas, M. *J. Org. Chem.* **1995**, *60*, 7017-7021.
7. Khodair, A. I.; Al-Masoudi, N. A.; Gesson, J.-P. *Nucleos. Nucleot. Nucl.* **2003**, *22*, 2061-2076.
8. Pozsgay, V.; Jennings, H. J. *J. Org. Chem.* **1988**, *53*, 4042-4052.
9. Komba, S.; Shiro, I.; Hideharu, K.; Kiso, M.; Hasegawa, A. *Bioorg. Med. Chem.* **1996**, *4*, 1833-1848.

10. Oturam, M. A.; Medebielle, M.; Patil, S. A.; Klein, R. S. *Turk. J. Chem*. **2002**, *26*, 317-322.
11. Tropper, F. D.; Andersson, F. O.; Grand-Maitre, C.; Roy, R. *Synthesis* **1991**, 734-736.
12. Zhu, X.; Dere, R. T.; Jiang, J.; Zhang, L.; Wang, X. *J. Org. Chem*. **2011**, *76*, 10187-10197.
13. Cerny, M.; Stanek, J.; Pacak, J. *Monatsh. Chem*. **1963**, *94*, 290-294.
14. Fiore, M.; Marra, A.; Dondoni, A. *J. Org. Chem*. **2009**, *74*, 4422-4425.
15. Ponpipom, M. M.; Bugianesi, R. L.; Blake, T. J. *J. Med. Chem*. **1987**, *30*, 705-710.
16. Meng, X.-B.; Yang, L.-D.; Li, H.; Li, Q.; Cheng, T.-M.; Cai, M.-S.; Li, Z.-J. *Carbohydr. Res*. **2002**, *337*, 977-981.
17. Fujihira, T.; Chida, M.; Kamijo, H.; Takido, T.; Seno, M. *J. Carbohydr. Chem*. **2002**, *21*, 287-292.

NMR spectra of new compounds:

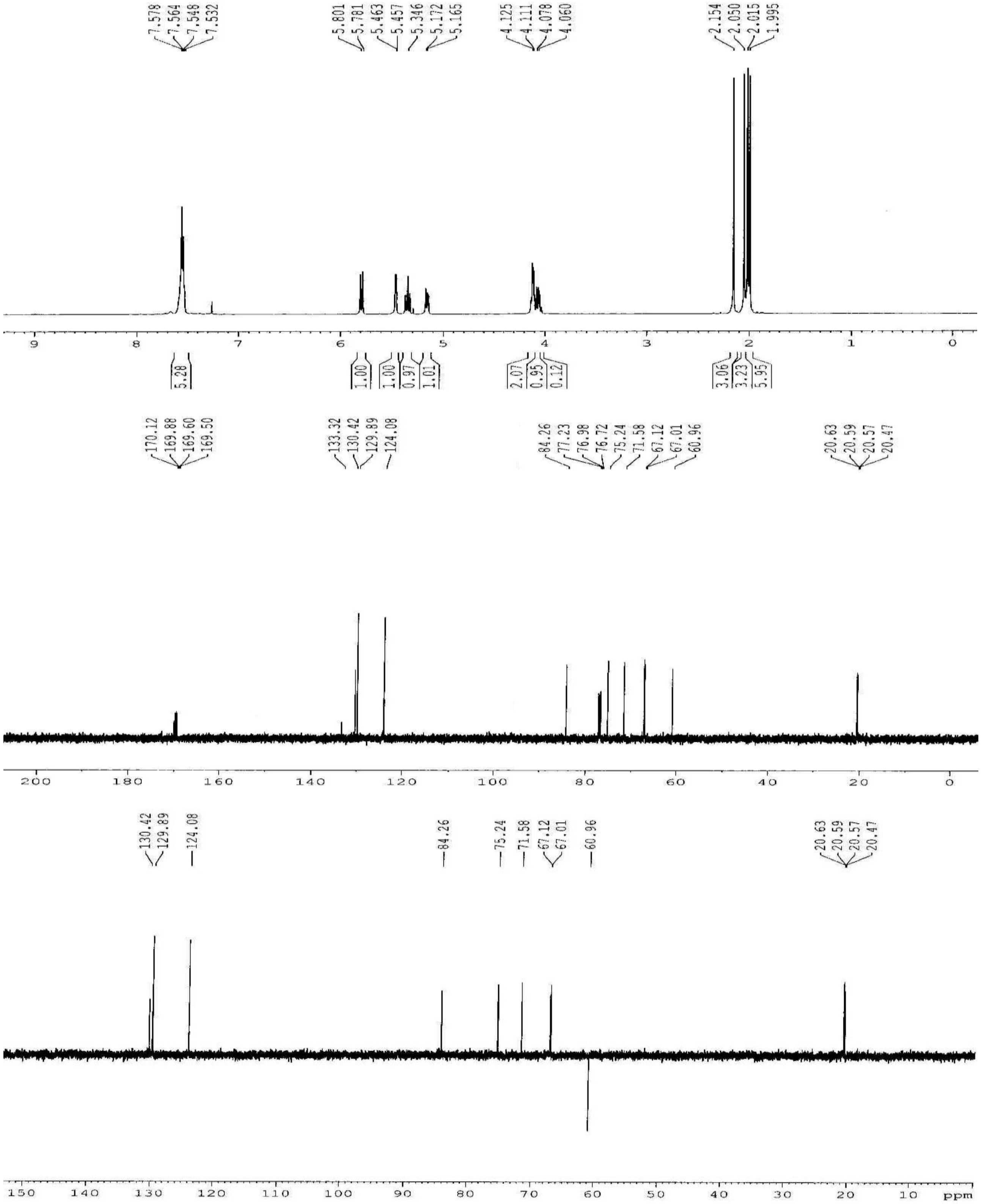

Figure S1: ^{1}H, ^{13}C and ^{13}C DEPT 135 NMR spectra of 1-phenyl-1*H*-tetrazol-5-yl 2,3,4,6-tetra-*O*-acetyl-1-thio-β-D-galactopyranoside **(17).**

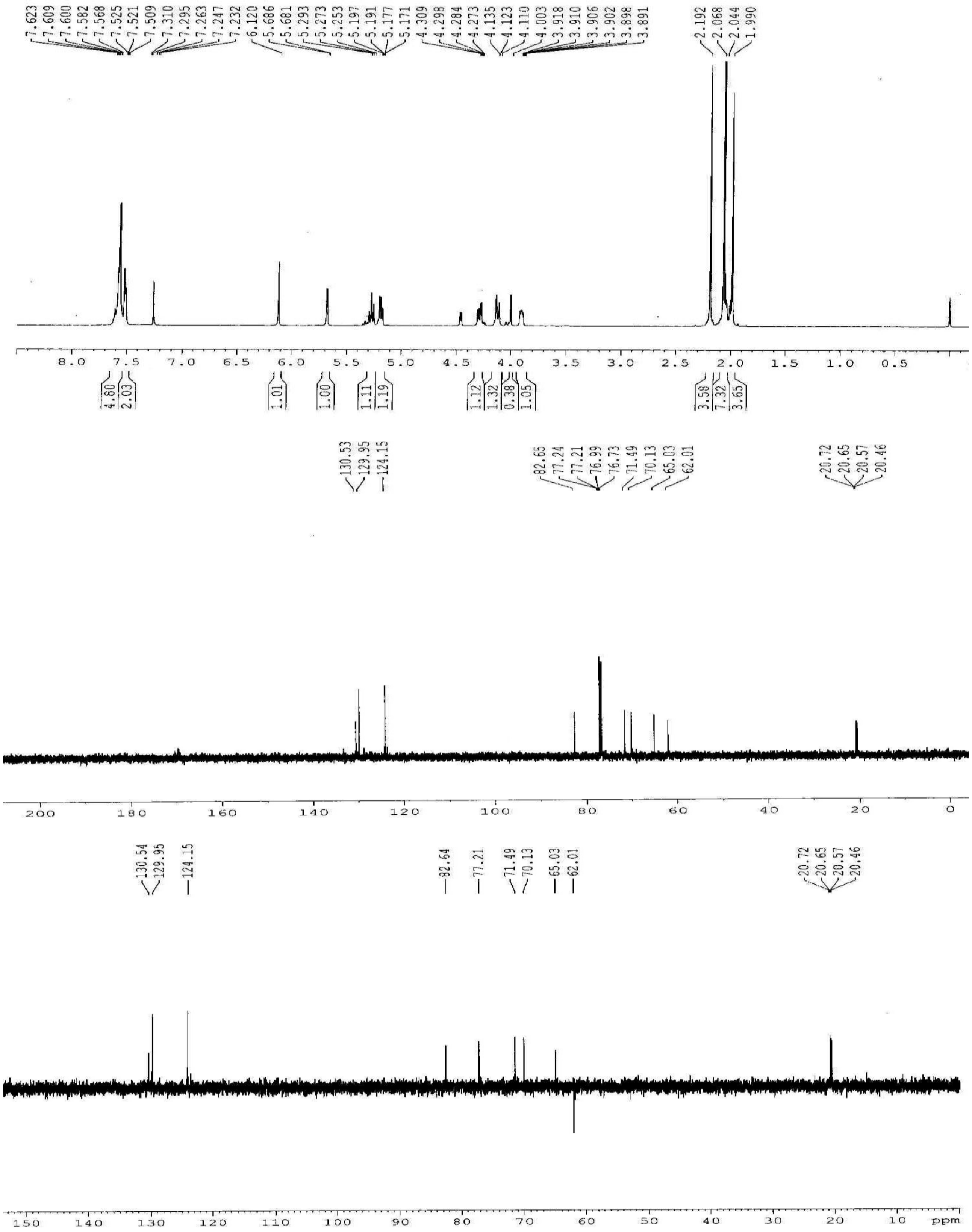

Figure S2: ^{1}H, ^{13}C and ^{13}C DEPT 135 NMR spectra of 1-phenyl-1*H*-tetrazol-5-yl 2,3,4,6-tetra-*O*-acetyl-1-thio-α-D-mannopyranoside **(20)**.

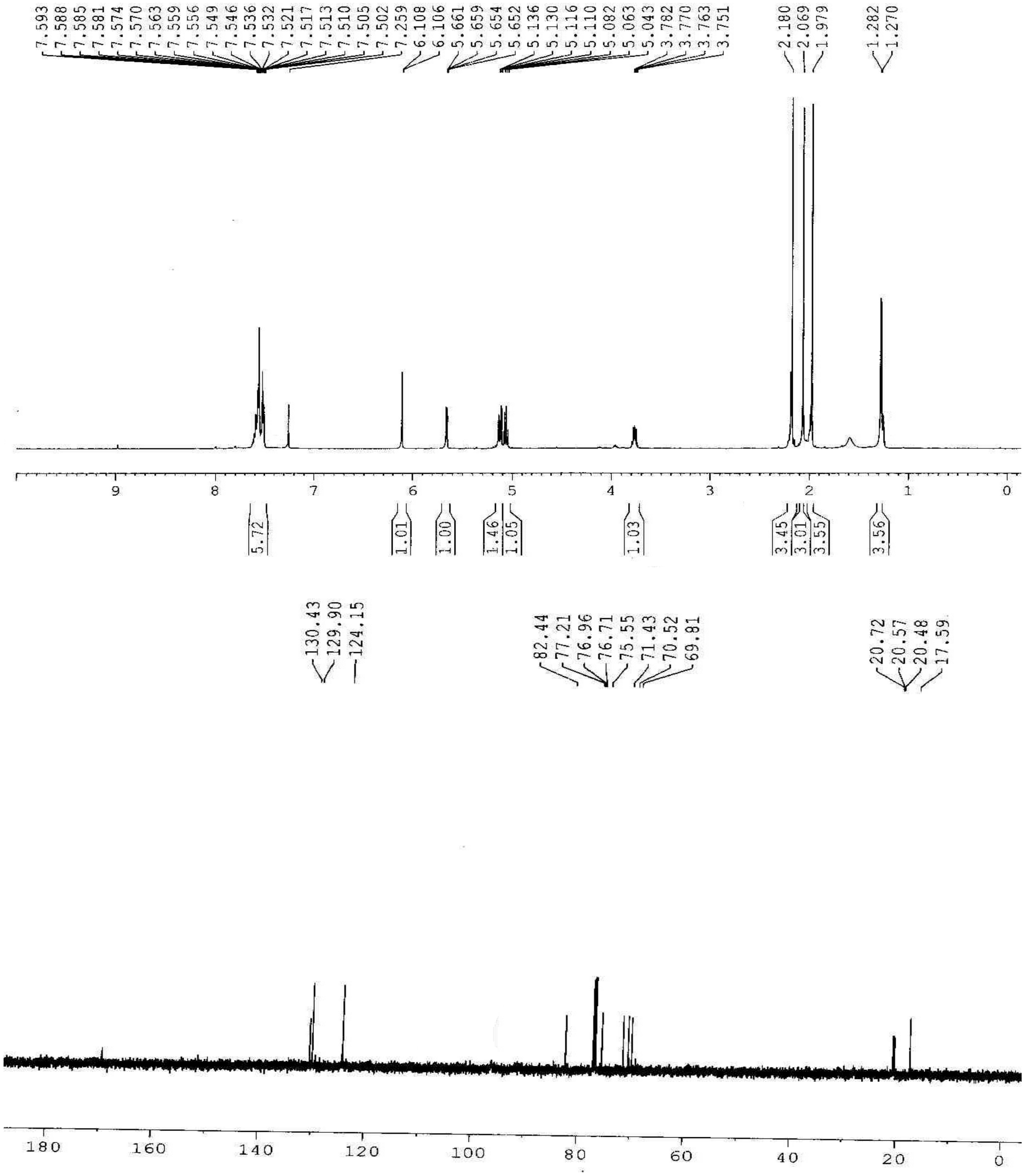

Figure S3: ^{1}H, ^{13}C and ^{13}C DEPT 135 NMR spectra of 1-phenyl-1*H*-tetrazol-5-yl 2,3,4-tri-*O*-acetyl-1-thio-α-L-rhamnopyranoside **(23).**
